# Identification of potential hub genes and molecular mechanisms in breast cancer microenvironment: A comprehensive transcriptomics approach

**DOI:** 10.1097/MD.0000000000044142

**Published:** 2025-08-29

**Authors:** Abdullah Al Noman, Abdullah Al Saba, Mohammad Sayem, Tahirah Yasmin, A. H. M. Nurun Nabi

**Affiliations:** a Department of Biochemistry and Molecular Biology, Laboratory of Population Genetics, University of Dhaka, Dhaka, Bangladesh.

**Keywords:** breast cancer, differentially expressed genes, gene enrichment, hub genes, transcriptomics

## Abstract

Breast cancer, a major health concern worldwide, involves diverse molecular subtypes and complex gene expression patterns. This study conducted a comprehensive bioinformatics analysis of breast cancer, analyzing 10 gene expression datasets from the Gene Expression Omnibus archive to find common genes that exhibit differential expression (DEGs). Then, we conducted pan-cancer and functional enrichment analyses, including single-cell level investigations of DEGs. To identify potential hub genes, we built protein–protein interaction networks, which were further analyzed for methylation patterns and expression in various cancer stages. The study also explored interactions between these hub genes with microRNAs and transcription factors, along with their correlation with cytokine expression and infiltration of immune cells in the tumor microenvironment. The TCGA BRCA dataset was used for hub gene survival analysis. Key findings include the identification of 36 shared DEGs, with distinct expression patterns in principal component analysis, suggesting their potential as molecular signatures for diagnosis and prognosis. Investigation at the single-cell level revealed upregulated DEGs mainly expressed by tumor-associated fibroblast cells. Gene ontology unveiled upregulated genes mainly involved in the extracellular matrix, but most of the downregulated genes are tumor suppressors. The protein–protein interaction network analysis highlighted 7 hub genes: *CAV1*, *FN1*, *BGN*, *CXCL12*, *SPTBN1*, *COL11A1*, and *INHBA*. Methylation analysis revealed intricate epigenetic regulation of these genes. MicroRNA and transcription factor interaction analysis underscored the complex regulatory networks influencing gene expression. Cytokine profile analysis in tumor microenvironment and its correlation with hub gene expression provided insights into the immunological landscape of breast cancer. Survival analysis indicated that both upregulated and downregulated hub genes are linked with patient overall survival outcomes, although it is not statistically significant. This study provides a detailed view of the underlying molecular mechanisms in breast cancer, suggesting potential biomarkers and therapeutic targets. Its findings can contribute significantly to understanding the complexity of the breast cancer microenvironment, leading to the development of more sophisticated and targeted treatment strategies.

## 1. Introduction

Breast cancer represents a significant public health concern and a major cause of premature mortality in females. It remains to be the most predominant type of cancer in women globally, representing 25% of total cancer diagnoses and 15% of cancer deaths.^[1]^ In the USA, an estimated 300,590 cases of breast cancer were diagnosed, and about 43,170 women died from the disease in 2023.^[1]^ By 2040, it is anticipated that there would be more than 40% more new cases and 50% more deaths from breast cancer, mostly as a result of aging and population growth.^[2]^ Factors such as family history, genetics, aging process, reproductive issues, obesity, alcohol intake, estrogen exposure, and physical activity can raise the chance of developing breast cancer.^[3–5]^ Along with these, carcinogenic components, free radicals, ionizing radiation, heterocyclic aromatic amines also contribute to the development of breast cancer.^[6]^ These factors may have different effects on the risk of different breast cancer subtypes.^[7]^

Four distinct subtypes of breast cancer can be classified according to gene expression patterns: basal-like, luminal A, luminal B, and HER2-enriched.^[8]^ The subtypes have different prognoses, responses to treatment, and survival outcomes. Luminal A has high estrogen receptor (ER) and low Ki-67 and HER2 expression, and has the best prognosis and response to endocrine therapy.^[9]^ Luminal B has high ER and high Ki-67 and/or HER2 expression and has a worse prognosis and may need anti-HER2 therapy and chemotherapy.^[9]^ HER2-enriched has high HER2 and low ER and progesterone receptor expression and has a poor prognosis but can be treated with anti-HER2 therapy and chemotherapy.^[10]^ Basal-like has low ER, progesterone receptor, and HER2 expression and high CK5/6 and/or epidermal growth factor receptor expression, and has the worst prognosis and is often associated with BRCA1 mutations.^[9]^ RNA-seq and microarray analysis complement each other, and recently, in the field of cancer research, there has been growing interest in combining microarray analysis with high-throughput sequencing data using bioinformatics techniques.^[11]^ Previous studies often focused on specific subtypes of breast cancer or compared breast tumor tissues to normal tissues.^[12,13]^ While informative, this approach may not capture the full range of molecular differences among breast cancer types. Additionally, previous studies sometimes lacked validation for RNA sequencing data and had small sample sizes, which can introduce bias and limit the generalizability of their findings.^[14]^ While bulk gene expression analysis offers overall trends, most of such studies do not validate these findings to the single-cell level. This validation is crucial for understanding the transcriptional landscape of various cell types present in the microenvironment of the tumor. Moreover, in addition to gene expression profiling, transcription factors (TFs) and microRNAs (miRNAs) have been increasingly recognized as important biomarkers in various cancers.^[15,16]^

In this study, we aimed to conduct an extensive bioinformatics analysis to provide a more precise view on the expression landscape of breast cancer. We acquired 10 gene expression profiles of breast cancer encompassing all major subtypes from the Gene Expression Omnibus (GEO) database. Then we analyzed the datasets to find out genes that are differentially expressed and performed functional enrichment analysis of these shared differentially expressed genes (DEGs) through Gene Ontology (GO) and Kyoto Encyclopedia of Genes and Genomes (KEGG). To elucidate the specific cellular origins of these DEGs, we explored their expression in single-cell RNA sequencing datasets encompassing various cell types that constitute the intricate landscape of the breast cancer tumor microenvironment (TME). Furthermore, protein–protein interaction (PPI) networks were constructed for the DEGs, which facilitated the identification of hub genes. Subsequently, we analyzed the methylation landscape of the hub gene promoters and explored the dynamics of their expression in different stages of cancer progression. In addition to this, we delved into the intricate network of interactions involving TFs and miRNAs with the identified hub genes. We also examined the relationship of immune cell infiltration and cytokine expression with hub gene expression. Finally, we performed survival analysis based on the expression status of hub genes within the TCGA BRCA dataset, to gain insights into the potential impact of hub gene expression on patient survival outcomes. This comprehensive investigation is expected to yield valuable insights into the complex molecular processes driving cancer progression and uncovering new possibilities for the development of therapeutic targets.

## 2. Methods

### 2.1. Ethics statement

Ethical approval was not required for this study because all datasets were obtained from a publicly accessible Database (https://www.ncbi.nlm.nih.gov/geo/).

### 2.2. Data acquisition

In this study, 10 microarray datasets of breast cancer were collected from the GEO database (https://www.ncbi.nlm.nih.gov/geo/). GEO is an open-source genomics data archive that contains array and sequence-based data.^[17]^ The selected datasets met the following criteria: datasets with tissue samples taken from people with breast cancer and corresponding normal tissue; patients with no therapy or treatment; the UMAP visualization of the breast cancer and normal samples exhibit distinct clusters. Detailed information on the datasets used in this study is listed in Table 1.

**Table 1:** Microarray datasets utilized in this study.

Dataset	Number of samples		Experiment type	Array type
	Normal	Tumor		
GSE31448	4	353	Microarray	Affymetrix Human Genome U133 Plus 2.0 Array
GSE42568	17	104	Microarray	Affymetrix Human Genome U133 Plus 2.0 Array
GSE26910	6	6	Microarray	Affymetrix Human Genome U133 Plus 2.0 Array
GSE5364	13	183	Microarray	Affymetrix Human Genome U133A Array
GSE50428	5	26	Microarray	Agilent-021412 nONCOchip_1.0 021253
GSE29431	12	54	Microarray	Affymetrix Human Genome U133 Plus 2.0 Array
GSE10810	27	31	Microarray	Affymetrix Human Genome U133 Plus 2.0 Array
GSE65216	11	153	Microarray	Affymetrix Human Genome U133 Plus 2.0 Array
GSE45827	11	130	Microarray	Affymetrix Human Genome U133 Plus 2.0 Array
GSE21422	5	14	Microarray	Affymetrix Human Genome U133 Plus 2.0 Array
Total	111	1054		

### 2.3. DEGs analysis

To identify DEGs, 10 microarray datasets were examined utilizing the web program GEO2R (http://www.ncbi.nlm.nih.gov/geo/geo2r/). GEO2R analyzes 2 or more sample groups in a GEO data series to find genes that are differently expressed.^[18]^ For microarray data analysis, it employs several R tools from the Bioconductor project, including GEOquery and limma. To identify the significant DEGs, we set a threshold: |log_2_ FC| >1 and adjusted *P* value < .05. Mutual DEGs were visualized in an upset plot using an R package UpSetR^[19]^ The log fold change of shared DEGs was then used to generate a heatmap using the pheatmap package in R.

### 2.4. Principal component analysis (PCA) of shared DEGs in the TCGA BRCA dataset

PCA is a dimensionality reduction method used to minimize the number of variables in large data sets while preserving the bulk of the information in the larger set. To distinguish between healthy persons and breast cancer patients based on the DEGs, PCA was conducted using the Gene Expression Profiling Interactive Analysis 2 (GEPIA2) website (http://gepia2.cancer-pku.cn/#index). GEPIA2 is a repository for gene expression analysis using samples from both normal and cancerous tissues sourced from GTEx and TCGA databases. The Cancer Genome Atlas Program TCGA is an open-source database that contains information on over 20,000 primary cancers and paired normal samples encompassing 33 types of cancer. Genotype-Tissue Expression is a freely accessible resource for exploring tissue-specific gene expression and regulation.^[20]^ Genotype-Tissue Expression was employed to enhance the size of the sample of healthy tissues.

### 2.5. Pan-cancer analysis of shared DEGs expression

Multiple gene expression comparison features in GEPIA2 were employed to analyze common DEGs in 30 cancers and the equivalent normal tissues. This tool creates a graphical representation of expression matrix data based on a provided gene list. When normalized by the highest median expression value across all blocks, the color intensity in every block indicates the value of the median expression of a gene in a particular tissue. The data was retrieved from GEPIA2, and a customized heatmap was generated using the R language.

### 2.6. Functional enrichment analysis of the shared DEGs

The gene set enrichment analysis of upregulated and downregulated genes, encompassing molecular functions (MF), cellular components (CC), biological processes (BP), and KEGG pathways was conducted employing the ClusterprofileR package in the R programming language.^[21]^ Identifying the biological features that are most distinctive in high-throughput sequencing data requires performing a pathway enrichment study.

### 2.7. Single-cell RNA sequencing investigation of shared DEGs

To investigate common DEGs in a single-cell level, the single-cell portal (SCP) (https://singlecell.broadinstitute.org/single_cell) was employed. SCP is a scalable, cloud-based web tool that is categorized by studies and offers raw data for download, which allows repeatable single-cell research rapidly. We conducted a search for information related to “breast cancer” within the SCP database. Among the findings, we identified a dataset^[22]^ encompassing the genetic profiles of 100,064 cells originating from breast cancer tissues, including several subtypes of breast cancer cells, cancer-associated fibroblast cells, endothelial cells and immune cells.

### 2.8. Exploring PPI

The NetworkAnalyst v3.0 web tool (https://www.networkanalyst.ca/) was utilized to build the PPI network of shared DEGs. The network analysis, meta-analysis, and transcriptome profiling capabilities of NetworkAnalyst make it a cutting-edge visual analytics tool. The STRING interactome database was selected after the submission of a common gene symbol, and the cutoff confidence score was set at 900. The hub protein network was then built using the networks that had been modified using Cytoscape v3.10. Cytoscape is a publicly available software platform designed for depicting networks of molecular interaction and biological pathways, which has some additional features that are available as Plugins.

### 2.9. Hub genes identification

In networks of genes, a hub gene refers to a gene that has numerous connections with other genes. These hub genes are crucial in regulating genes and driving various BP.^[23]^ To identify the hub genes, the Degree algorithm in the cytohubba (v0.1) plugin in Cytoscape v3.10 was used. The hub genes with scores ≥ 10 were selected for downstream analysis.

### 2.10. Analysis of methylation level and hub genes expression in different stages

An assessment of methylation level disparities between healthy and cancerous tissue was executed utilizing the TCGA BRCA cohort on The University of Alabama at Birmingham CANcer data analysis Portal (UALCAN) server (https://ualcan.path.uab.edu/). This server was also selected to examine the expression of hub genes across various cancer stages. The hub genes were uploaded to the database, and the expression of each gene was observed. UALCAN serves as an interactive online platform designed for the comprehensive analysis of cancer OMICS data. This resource facilitates the convenient exploration of publicly accessible cancer OMICS datasets, including TCGA and CPTAC. Users can seamlessly pinpoint biomarkers and conduct in silico validation of potential genes. Moreover, UALCAN enables the assessment of gene expression’s epigenetic control via promoter methylation and offers pan-cancer gene expression analysis capability.

### 2.11. Exploring TF–hub genes and miRNA–hub genes interactions

The relationship between TF–genes and miRNA–genes was investigated via the NetworkAnalyst 3.0 server. The genes were uploaded and the TarBase8.0 database was chosen in the gene–miRNA interaction program for the miRNA–hub gene interaction. TarBase is a specialized database that compiles and presents a meticulously curated assortment of experimentally validated miRNA targets. Similarly, for the TF–hub genes interactions, the JASPAR database was used in the TF–hub genes interaction program. JASPAR is an openly available database characterized by its manual curation of unique transcription factor binding profiles spanning 6 taxonomic categories.

### 2.12. Cytokine expression in TME and correlation between hub gene with significant cytokine

To construct a cytokine expression profile in the breast cancer microenvironment, the previously used single-cell RNA sequencing dataset on the SCP was exploited. Twenty-four major cytokine gene symbols were uploaded to the portal, and the server generated a dot plot. Substantial expression of interleukin (IL)-1B, IL-6, IL-33, IL-18, TGF-B, tumor necrosis factor (TNF), and interferon-gamma (IFNG) in different cells in TME was observed, and finally, the correlations between these cytokines with the hub genes were found using TIMER2.0 (Dana Farber Cancer Institute, Boston) and a heatmap was constructed.

### 2.13. Correlation between hub gene and immune cell filtration

Immune cell infiltration is a crucial process by which various immune cells migrate from the bloodstream into the TME. The association between hub gene expression and immune infiltration of natural killer cells, macrophage, neutrophils, B-cell, CD4 + T-cell, and CD8 + T-cell was observed using the TIMER algorithm in TIMER2.0 server, then customized heatmap was generated using R language. TIMER utilizes linear least square regression algorithm to evaluate the quantity of 6 types of tumor infiltrating cells.^[24]^

### 2.14. Survival analysis based on expression status of hub gene

Survival analysis primarily focuses on studying the time until events like patient deaths or disease progression in cancer. The hub genes were entered into the GEPIA2 tool, and overall survival (OS) was performed in the TCGA BRCA dataset. To divide the cohorts with low and high expression, the median (50–50) group cutoff was selected. Samples exceeding this threshold in expression levels are classified as the high-expression group, while those falling below it are recognized as the low-expression group. It generated a Kaplan–Meier survival plot as well as performed a Mantel–Cox test (Log-rank test). The Kaplan–Meier survival curve is a graphical representation that shows the estimated probability of an event not occurring (usually survival) over a specified time. The Mantel–Cox test is a statistical method employed in survival analysis to assess if there are notable distinctions between the survival curves of 2 or more groups.

## 3. Results

**Figure 1.**
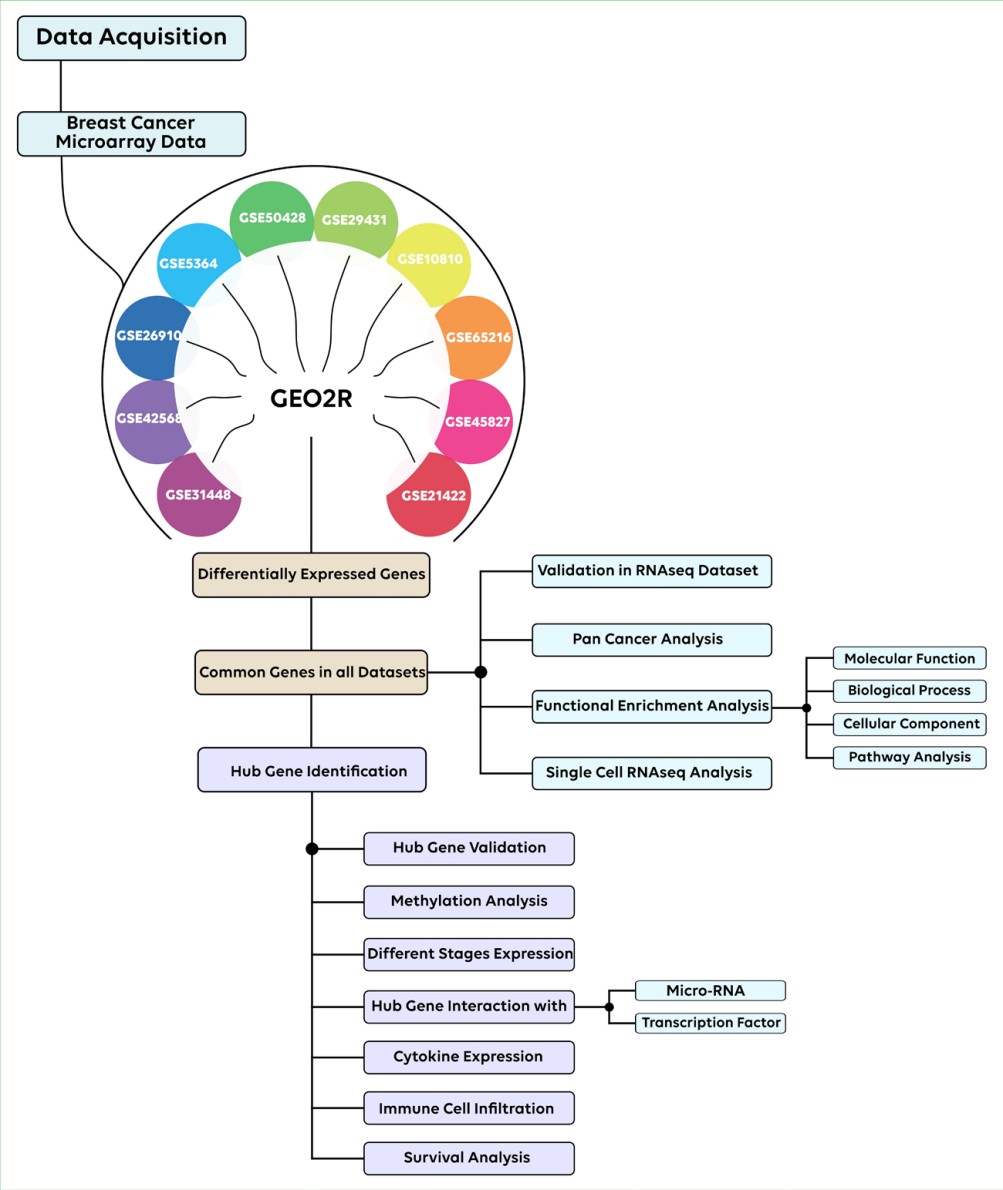
Workflow of the study.

Fig. 1 depicts the entire organizational structure of the study. Table 1 lists the datasets along with the experimental data.

### 3.1. DEGs identification and validation

**Figure 2.**
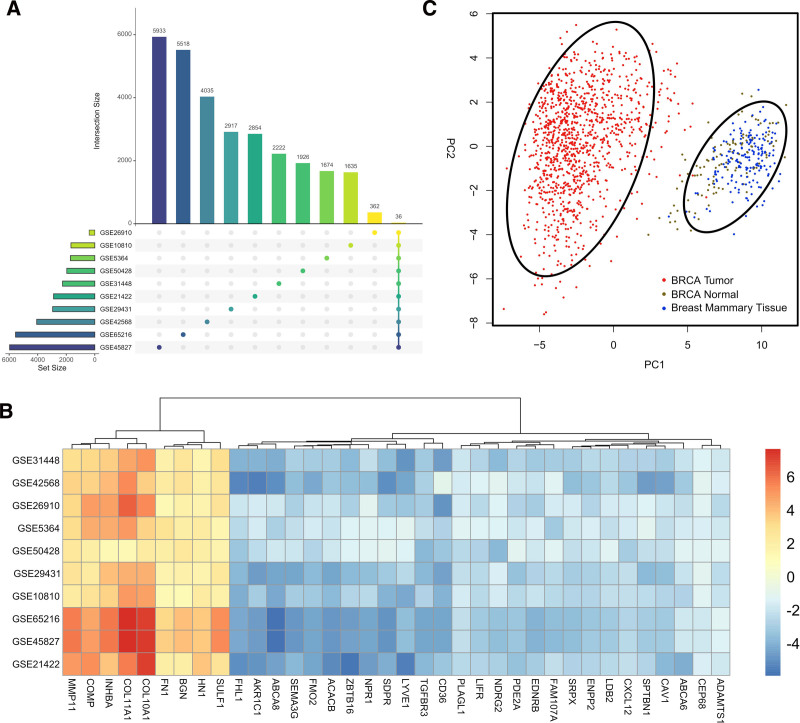
(A) The UpSet plot displays the total number of genes across all datasets that are differentially expressed. Across all datasets, 36 common genes are identified. (B) A heatmap of expression data based on log2 fold change for common differentially expressed genes (DEGs) in all datasets is displayed. Genes that are upregulated in breast cancer are shown in red, whereas those that are downregulated are represented in blue. Among the 36 shared DEGs, 9 genes are upregulated in all datasets, and the rest are downregulated. (C) Principal component analysis (PCA) of DEGs. Red color indicates BRCA tumor samples, green color indicates BRCA normal samples, and blue color indicates breast mammary tissues. In the PCA plot, both tumor and normal tissues show distinct clusters.

Total 2222, 4035, 362, 1674, 1926, 2917, 1635, 5518, 5933, and 2854 DEGs were found in GSE31448, GSE42568, GSE26910, GSE5364, GSE50428, GSE29431, GSE10810, GSE65216, GSE45827, and GSE21422 datasets, respectively. The number of DEGs shared by 10 breast cancer datasets is displayed in the upset plot in (Fig. 2A). A total of 36 shared DEGs were found in all datasets. Among these DEGs, 27 genes were downregulated; they are *CEP68*, *ADAMTS1*, *PLAGL1*, *ABCA6*, *NPR1*, *ENPP2*, *LDB2*, *LIFR*, *PDE2A*, *CXCL12*, *SPTBN1*, *SEMA3G*, *SRPX*, *FAM107A*, *CAV1*, *FMO2*, *ACACB*, *EDNRB*, *TGFBR3*, *SDPR*, *NDRG2*, *ZBTB16*, *AKR1C1*, *FHL1*, *ABCA8*, *CD36*, and *LYVE1.* On the other hand, 9 genes were elevated in breast cancer; they are *COL10A1*, *COL11A1*, *INHBA*, *COMP*, *SULF1*, *MMP11*, *BGN*, *FN1*, and *HN1*. The heatmap in (Fig. 2B) displays the common 36 DEGs from gene expression datasets, along with their relationships, using a log2 fold change. From the pattern of the expression of these 36 DEGs, 2 different clusters are observed in the PCA analysis, from which the breast cancer patients in the TCGA dataset could be efficiently differentiated from healthy individuals (Fig. 2C).

### 3.2. Pan-cancer analysis of common DEGs expression

**Figure 3.**
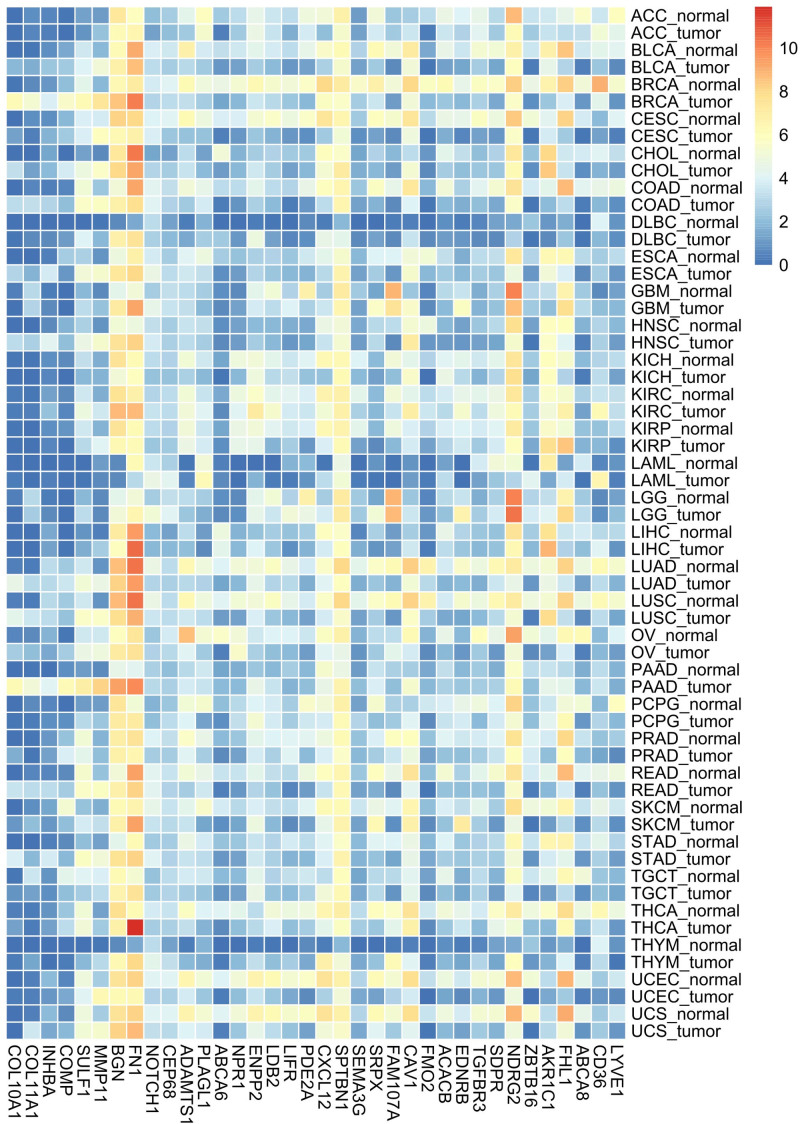
Pan-cancer analysis of common DEGs expression. The most upregulated genes are highly expressed in breast cancer and pancreatic ductal adenocarcinoma. Majority of downregulated genes expressed differentially in different cancers. DEGs = differentially expressed genes.

Pan-cancer analysis reveals that the most upregulated genes are prominent in breast cancer and pancreatic ductal adenocarcinoma (Fig. 3). These 2 types of cancer tissues show a common pattern of gene expression. The majority of downregulated genes exhibit differential expressions across various types of cancer tissues.

### 3.3. GO and KEGG pathway enrichment analysis

**Figure 4.**
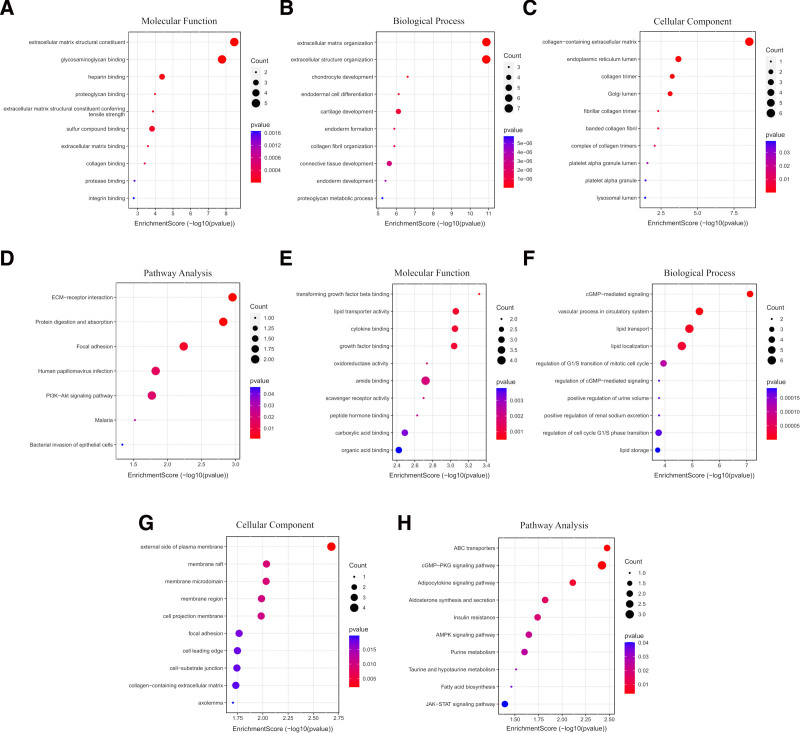
Functional enrichment analysis of upregulated and downregulated DEGs. (A–D) The enriched GO terms for molecular function, biological process, cellular components, and KEGG pathway of upregulated differentially expressed genes. (E–H) The enriched GO terms for downregulated differentially expressed genes, covering the same categories. The intensity of the red color indicates lower *P*-values and higher significance. DEGs = differentially expressed genes, GO = Gene Ontology, KEGG = Kyoto Encyclopedia of Genes and Genomes.

The upregulated genes were found to be abundant in MFs involving extracellular matrix structural constituent and binding of various types of molecules such as glycosaminoglycan, heparin, proteoglycan, etc, as well as BPs such as extracellular matrix organization (Fig. 4A and B). The upregulated genes were also much abundant in the CC encompassing collagen-containing extracellular matrix, collagen trimer, Golgi lumen, and endoplasmic reticulum lumen (Fig. 4C). KEGG analysis revealed that upregulated genes are linked with protein digestion and absorption, extracellular matrix–receptor interaction, PI3K–Akt signaling pathway, and focal adhesion (Fig. 4D). The downregulated genes were discovered to be associated with MFs such as transforming growth factor beta binding, lipid transporter activity, cytokine binding, and growth factor binding (Fig. 4E), as well as essential BPs such as cGMP-mediated signaling, vascular processes in the circulatory system, lipid transport, lipid localization, and regulating the mitotic cycle’s G1/S transition (Fig. 4F). When it comes to CC, these downregulated genes were primarily abundant in the membrane microdomain, membrane raft, and outside of the plasma membrane (Fig. 4G). These downregulated genes are mostly in charge of the cGMP–PKG, adipocytokine, and ABC transporters signaling pathways, according to KEGG pathway analysis (Fig. 4H). Thus, these DEGs were recognized as essential by enrichment analysis.

### 3.4. Single-cell RNA sequencing analysis of common DEGs

**Figure 5.**
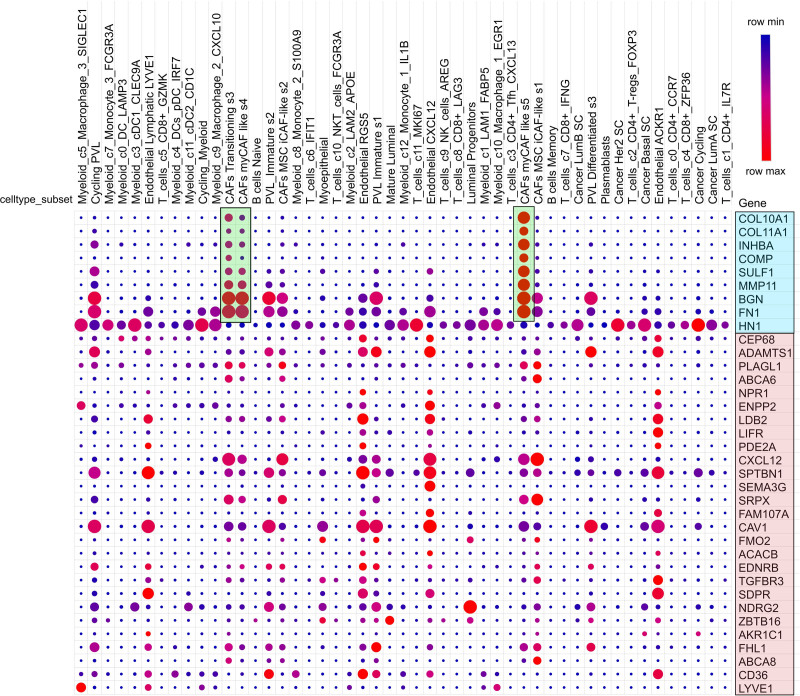
Single-cell RNA sequencing analysis of common DEGs. The light blue box indicates upregulated DEGs, while the light red color indicates downregulated DEGs. Light green color indicates that most upregulated genes are expressed in cancer-associated fibroblast cells (CAFs). However, downregulated genes are differentially expressed in different cells. DEGs = differentially expressed genes.

Most upregulated genes are expressed in cancer-associated fibroblast cells (Fig. 5). *BGN* and *FN1* genes are also significantly expressed in perivascular-like cells (PVL). *HN1* genes are noticeably expressed in cancer epithelial cells and myeloid cells like macrophages. In contrast, Downregulated genes are significantly downregulated in cancer-associated fibroblast cells but upregulated in different cell types. However, the overall expression is downregulated in bulk sequencing.

### 3.5. PPI network analysis and hub gene detection

**Figure 6.**
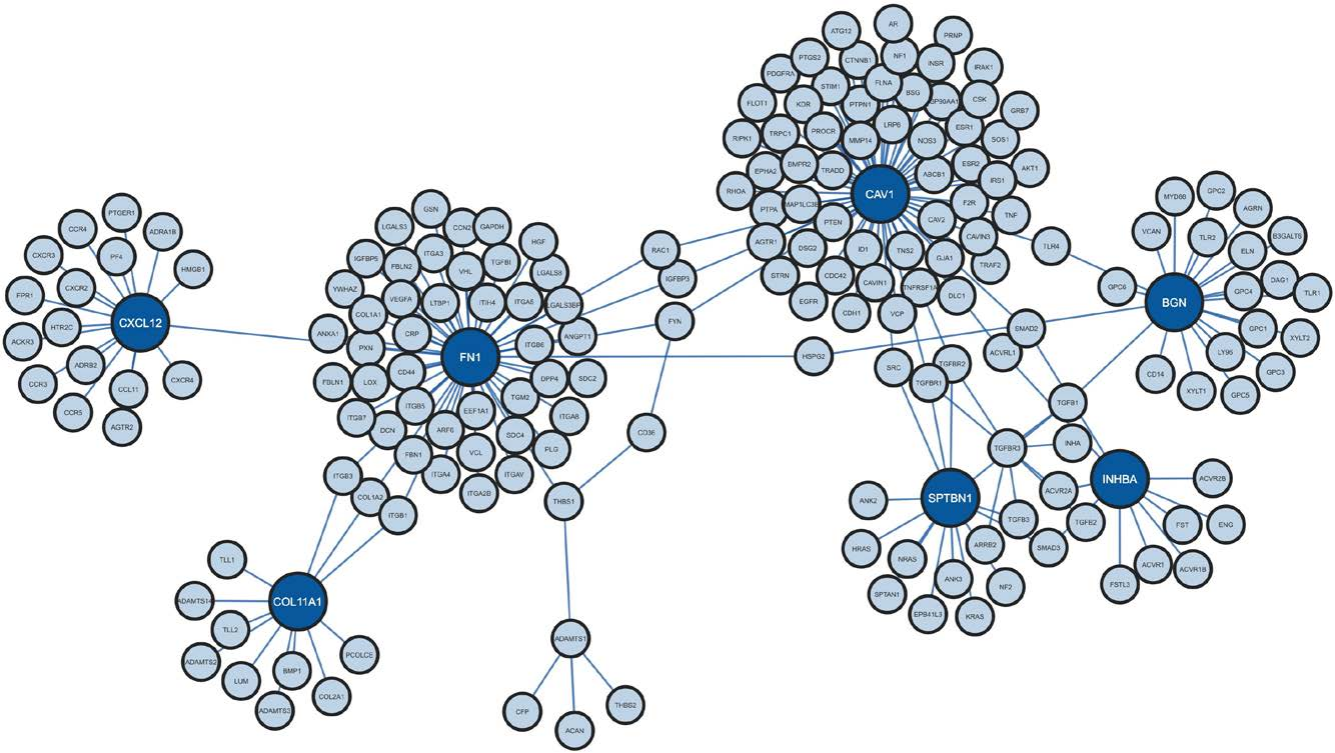
The protein–protein interaction network analysis using the Degree method. Dark blue color represents hub genes. The results indicate that 7 proteins: *CAV1*, *FN1*, *BGN*, *CXCL12*, *SPTBN1*, *COL11A1*, and *INHBA* have the potential to act as hub proteins.

The PPI network revealed 10 seeds, 188 nodes, and 202 edges (Fig. 6). The findings showed that seeds that might serve as hub proteins were *CAV1*, *FN1*, *BGN*, *CXCL12*, *SPTBN1*, *COL11A1*, *INHBA* (organized as descending order with respect to score). *FN1*, *BGN*, *COL11A1*, and *INHBA* are upregulated, and *CAV1*, *CXCL12*, and *SPTBN1* are downregulated hub genes. The hub proteins are listed in Table 2 by Degree method.

**Table 2:** The hub proteins determined by Degree method.

Top 7 hub gene ranked by Degree method				
Rank	Name	Gene name	Score	Expression in breast cancer
1	857	CAV1	64	Down
2	2335	FN1	51	Up
3	633	BGN	21	Up
4	6387	CXCL12	17	Down
5	6711	SPTBN1	14	Down
6	1301	COL11A1	12	Up
7	3624	INHBA	10	Up

### 3.6. Methylation analysis and hub gene expression in different stages

**Figure 7.**
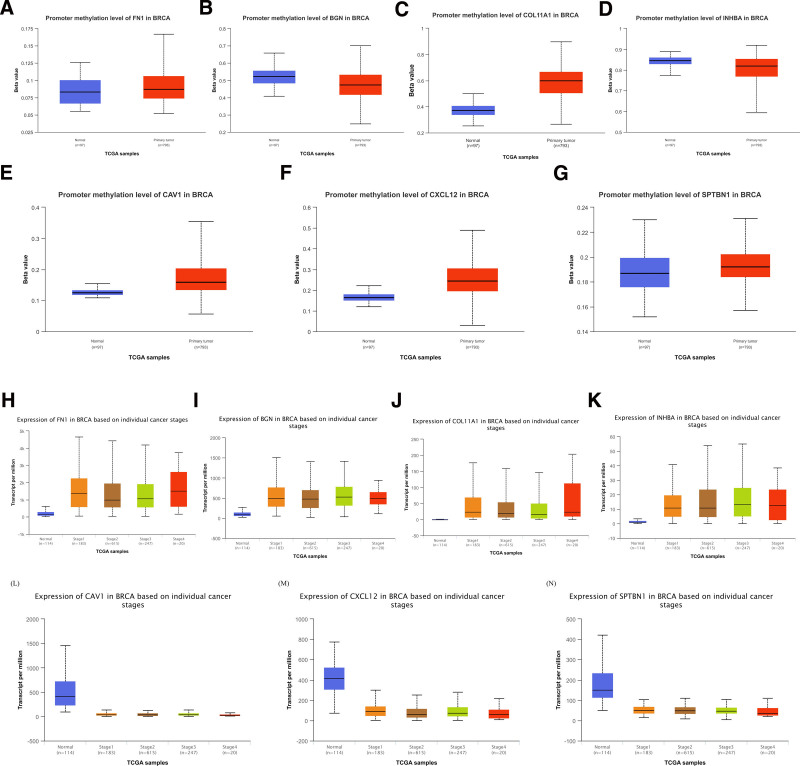
Hub genes promoter methylation and expression analysis. (A–G) The promoter methylation levels in breast cancer and normal tissues for FN1 (A), BGN (B), COL11A1 (C), INHBA (D), CAV1 (E), CXCL12 (F), and SPTBN1 (G) gene. The DNA methylation level is shown by the beta (β) value, which ranges between 0 (unmethylated) to 1 (completely methylated). Various cutoff values for β have been considered to indicate hypo (β value: 0.3–0.25) or hyper (β value: 0.7–0.5) methylated. (H–N) The expression level of hub genes by transcripts per million. No significant difference is found between different stages of cancer.

The promoter methylation of the downregulated hub genes is remarkably elevated (*P* < .05) compared to normal cells in cancer tissue. On the other hand, for upregulated hub genes, the methylation level was reduced in the case of *BGN* and *INHBA*. Unexpectedly, the methylation is notably increased in the case of *FN1* and *COL11A1* hub genes. However, these genes are upregulated in cancer cells (*P* < .05) (Fig. 7A–G). The hub gene expression level is relatively stable in all stages of cancer (Fig. 7H–N).

### 3.7. Exploring miRNA–hub genes and TF–hub genes interactions

**Figure 8.**
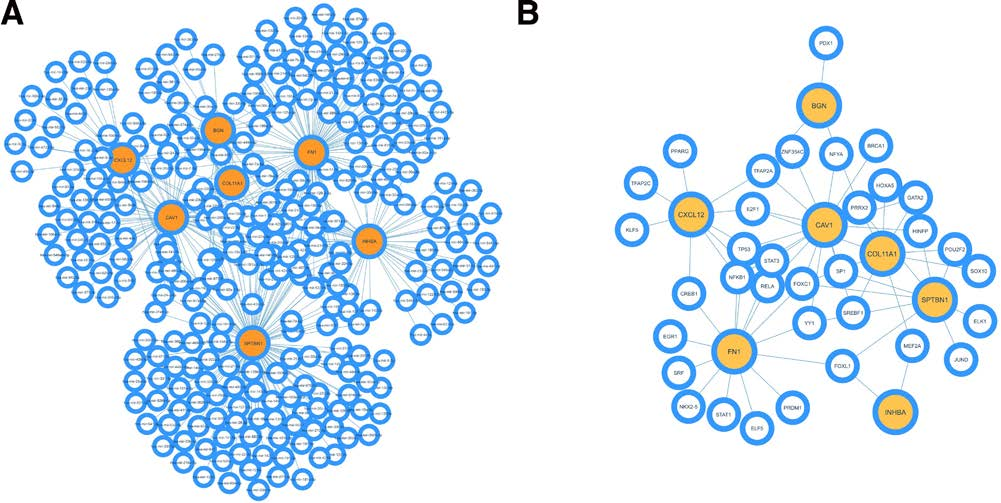
Interaction networks of hub genes with miRNA and transcription factors (TFs). In these networks, orange nodes represent hub genes, white nodes signify miRNAs (A), and TFs (B).

Total 288 miRNAs and 41 TFs were identified to interact with the hub gene. Among these 288 miRNAs, hsa-mir-1-3p was found to connect with 6 hub genes, while hsa-mir-16-5p and hsa-mir-27a-3p were observed to connect with 5 hub genes each (Fig. 8A). On the other hand, the network consisting of hub genes and transcription factors comprises 41 nodes and 62 edges. Among these 41 TFs, *FOXC1* displays the highest level of connectivity with the hub gene, with a connectivity of 4. Additionally, *PRRX2, FOXL1, YY1, STAT3*, and *E2F1* exhibit connections with 3 hub genes each (Fig. 8B).

### 3.8. Cytokine expression in TME and correlation between hub gene expressions with significant cytokine

**Figure 9.**
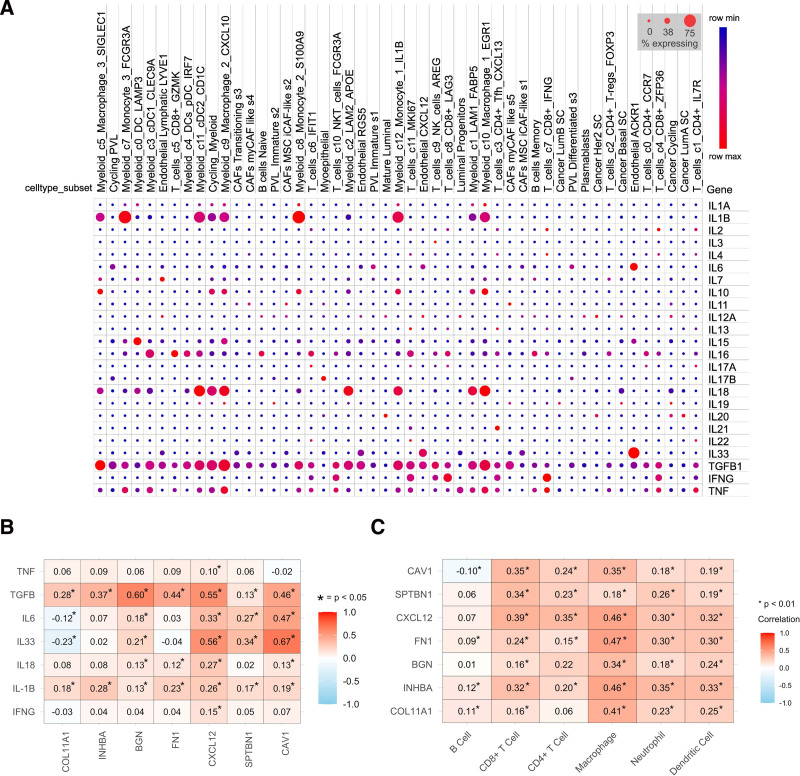
(A) Dot plot of cytokine expression in the tumor microenvironment at the single-cell level. Each dot represents the expression level, with larger dots indicating higher expression. (B) Correlation analysis of hub genes with significant cytokines (TNF, TGF-B, IL-6, IL-33, IL-18, IL-1B, and IFNG) found in single-cell investigations in breast cancer. The higher the intensity of the red color, the stronger the correlation. An asterisk (*) indicates statistically significant correlations. (C) The correlation analysis between the infiltration of immune cells in breast cancer tissues and the levels of hub genes *FN1*, *BGN*, *COL11A1*, *INHBA*, *CAV1*, *CXCL12*, and *SPTBN1*.

Out of the major 24 cytokines, IL-1B exhibits elevated expression in myeloid cells like monocytes and macrophages, whereas IL-33 and IL-6 are predominantly expressed in endothelial cells (Fig. 9A). In the TME, IL-18 and TGF-B are exclusively found in macrophages. Diverse immune cell types release TNF, while T cells uniquely secrete IFNG. The remaining cytokines, however, do not show substantial increases in expression levels. Figure 9B shows the association between hub gene expression with cytokines.

The expression of TGF-B1 and IL-1B is positively correlated with all hub genes, indicating their expression are independent from hub genes, whether they upregulated or downregulated. There is no correlation between TNF and IFNG with any hub genes. IL-6 and IL-33 show a strong positive correlation with downregulated hub genes but a negative correlation with *COL11A1*. This suggests that the upregulation of *COL11A1* and the downregulation of *CAV1*, *CXCL12*, and *SPTBN1* in the cancer microenvironment led to the downregulation of IL-6 and IL-33.

### 3.9. Correlation analysis between hub gene expression and immune cell infiltration

After analyzing the association between the immune cell infiltration levels and the expression levels of hub genes, it was observed that every hub gene exhibited a statistically significant positive correlation with a minimum of 5 immune cell infiltration levels (Fig. 9C).

### 3.10. Survival analysis based on expression status of hub gene

**Figure 10.**
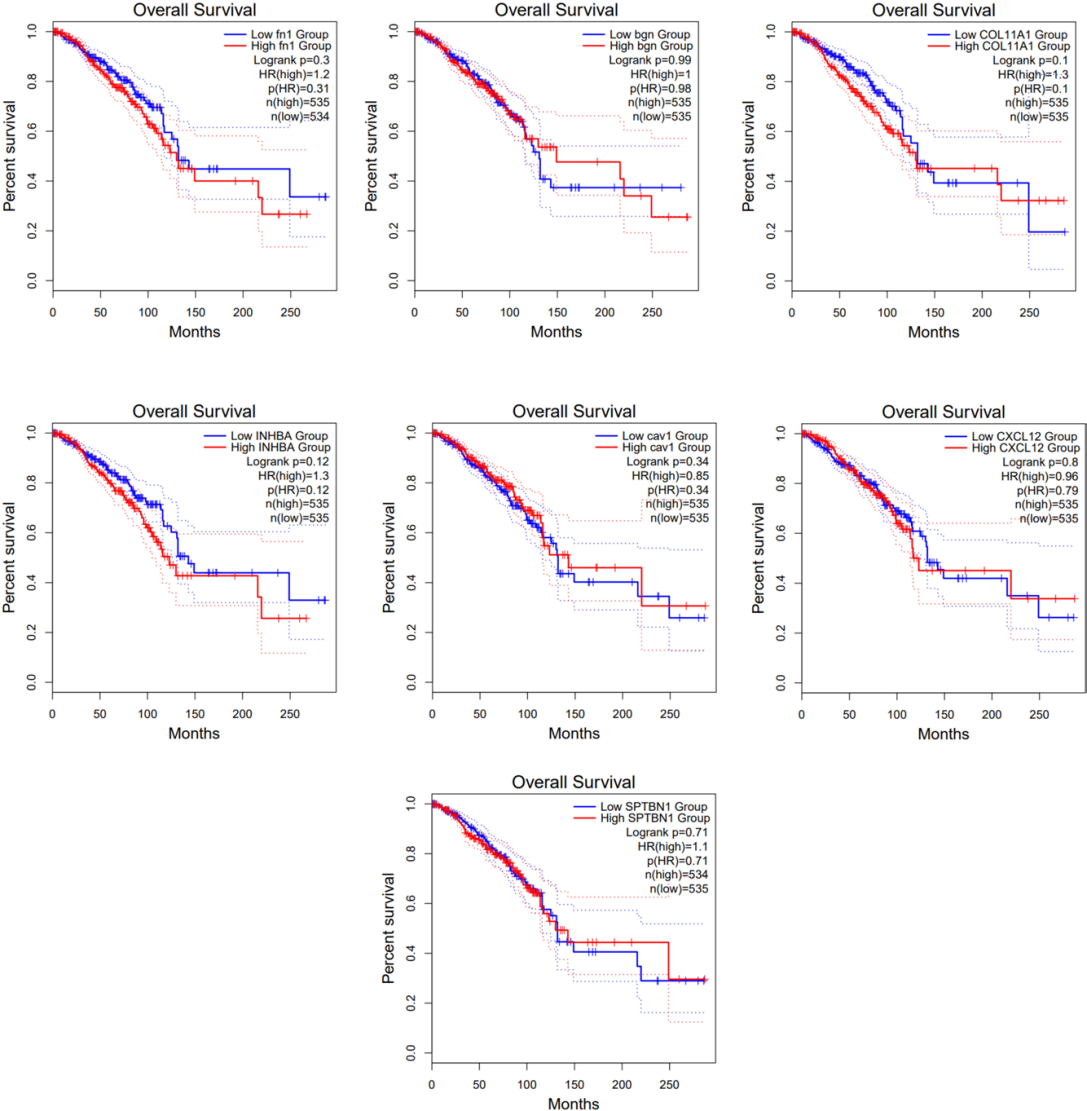
Overall survival analysis based on the gene expression status of hub genes. Samples in the TCGA BRCA dataset were divided into low and high-expression groups based on the median cutoff value. Samples with expression levels above this threshold are classified as the high-expression group, while those below it is considered as the low-expression group.

Survival analysis showed that higher levels of *BGN*, *FN1*, *COL11A1*, and *SPTBN1* are linked to poorer OS in patients with breast cancer. Conversely, reduced expression of *CXCL12*, *CAV1*, and *INHBA* genes is correlated with lower OS (Fig. 10) Although *P*-values for Log-rank were higher than .05.

## 4. Discussion

Breast cancer is a common and potentially fatal form of cancer, which is the most frequently diagnosed cancer among women globally.^[25]^ Breast cancer is a heterogenous condition where each patient shows unique characteristics, so researchers are looking for new markers to help with diagnosis, prognosis, and better treatment response.^[26]^ To identify enhanced diagnostic and therapeutic strategies, this study focused on revealing the DEGs associated with breast cancer and their possible implications in various aspects of the disease. This study provides a comprehensive insight into the molecular landscape of breast cancer through extensive bioinformatics analysis exploring differential gene expression, pan-cancer analysis, functional enrichment analysis, single-cell RNA sequencing analysis, PPI, epigenetic regulation, miRNA and TF interactions, and different correlation analysis to contribute to our understanding of breast cancer microenvironment collectively.

The analysis of 10 breast cancer microarray datasets revealed that 36 DEGs are present in all the datasets. This suggests the existence of common gene expression patterns associated with breast cancer across multiple studies. Most of the downregulated genes in this study are tumor suppressors. The commonly identified DEGs, whether upregulated or downregulated, have the potential to serve as biomarkers, therapeutic targets, and indicators of underlying mechanisms of initiation, progression, epithelial-mesenchymal transition, invasion and metastasis in different types of cancer.^[27–30]^ The pan-cancer analysis of common DEGs demonstrates diverse expression patterns in different cancers, requiring different treatment options for different cancers.^[31]^ Some intriguing similarities in upregulated gene expression patterns between breast cancer and pancreatic ductal adenocarcinoma. The consistency of gene expression patterns across these 2 distinct cancer types suggests a shared underlying mechanism or pathway that may be relevant to both cancers.

The GO and KEGG pathway analyses of upregulated and downregulated genes provided insights into their potential functions and implications. Upregulated genes were enriched in extracellular matrix and structure organization, indicating their involvement in cancer microenvironment reconditioning.^[32]^ Additionally, they were associated with essential CCs and pathways like focal adhesion, suggesting their roles in cancer cell proliferation, viability, adherence, movement, infiltration, and metastasis.^[33]^ The PI3K/AKT pathway is commonly overstimulated in various human cancers. This pathway influences a range of downstream target proteins, playing a significant role in cancer development, cell growth, invasion, and the spread of tumor cells.^[34]^ In contrast, downregulated genes were linked to vital BP and MFs, including cGMP-mediated signaling pathways, cytokine binding, lipid transport and localization, controlling the G1/S transition of the cell cycle indicating inhibit apoptosis of cancer cells and cancer development.^[35]^ The single-cell RNA sequencing provides valuable insights into cellular heterogeneity within the TME. Most of the upregulated genes were prominently expressed in cancer-associated fibroblast cells, not cancer cells, indicating their potential involvement in extracellular matrix remodeling, tumor cell proliferation, tumor progression and drug resistance.^[36]^ *HN1* genes are upregulated in cancer cells and contribute to breast cancer invasion, migration, and tumorigenesis by promoting MYC activity.^[37]^ On the other hand, most of the downregulated genes in this study are tumor suppressors, which is consistent with the literature.^[38]^ Therefore, the differential expression of genes in various cell types highlights the intricate crosstalk between cancer and normal cells, contributing to tumor heterogeneity.

PPIs are essential for cellular functions and BP and vital for developing new drugs and enhancing treatment strategies.^[39]^ Seven hub genes included both upregulated and downregulated genes, suggesting their regulatory significance in the disease. Understanding the roles of the hub genes, their interactions, and their implications in breast cancer progression can guide the development of targeted therapies. Upregulated hub genes could be targeted with specific inhibitors to impede their overactive functions in cancer cells. Conversely, downregulated hub genes might be candidates for gene therapy approaches or interventions to restore their normal expression levels. The examination of promoter methylation levels of hub genes revealed complex relationships between methylation and gene expression. All downregulated hub genes exhibited increased methylation levels. Upregulated hub genes *BGN* and *INHBA* exhibited reduced methylation levels, possibly contributing to their overexpression in cancer. Interestingly, *FN1* and *COL11A1*, despite being upregulated, displayed increased methylation levels. This paradox highlights the intricate epigenetic mechanisms that govern gene expression in cancer.^[40]^ The expression levels of the hub gene in all cancer stages are relatively stable. This stability in gene expression suggests that these hub genes maintain a consistent role throughout the development and progression of the cancer.

Gene expression can be regulated in different ways. TFs and miRNAs play crucial roles in controlling gene expression.^[41]^ TF and miRNA can control gene expression by altering the global epigenetic landscape of genes involved in different types of cancer.^[42]^ Regulation of TF and miRNA can be tumor-suppressive or carcinogenic and both are essential to understanding cancer initiation, progression, differentiation, and metabolism.^[43,44]^ Our study identified several transcription factors, such as *FOXC1, PRRX2, FOXL1, YY1, STAT3,* and *E2F1*, that exhibit substantial interactions with hub genes, potentially affecting their expression in breast cancer. *FOXC1* is involved in cancer cell proliferation, progression, differentiation, and metastasis in different cancer types, such as basal breast cancer, and hepatocellular carcinoma.^[45]^ *PRRX2* is linked with increased invasion and migration in mammary epithelial cells and is linked to unfavorable outcomes in breast cancer.^[46]^ In TME, STAT3 is overexpressed in both malignant and non-cancerous cells and inhibits the expression of immune activation.^[47]^ *E2F1* promotes cancer metastasis.^[48]^ This study identified various miRNAs associated with hub genes. hsa-mir-1-3p suppresses the proliferation of cancer cells in different cancers.^[49]^ has-mir-27a-3p inhibits tumor metastasis in hepatocellular carcinoma.^[50]^ hsa-mir-27b-3p suppresses hepatocellular carcinoma progression^[51]^ but promotes invasion and migration in colorectal cancer.^[52]^ hsa-mir-16-5p is associated with the progression of various tumors including osteosarcoma, cervical cancer, brain tumors, neuroblastoma, bladder cancer, lung cancer, breast cancer, gastrointestinal cancers, and hepatocellular carcinoma.^[53]^

Cancer cells, immune cells, and stromal cells secrete cytokines to promote processes that support tumor cell initiation, proliferation, immunosuppression, and metastasis.^[54]^ The cytokine expression profile in the TME provides insights into the immunological landscape of breast cancer and is crucial for effective drug targets. IL-1β promotes cancer cell migration, invasion, and aggressiveness while also inducing immunosuppression, local tumor growth, and angiogenesis.^[55]^ IL-33 might function as an anti-tumorigenic and pro-tumorigenic cytokine.^[56]^ Depending on the context, IL-6 can prevent or promote cancer development.^[57]^ IL-18 is a pro-inflammatory cytokine that is associated with tumor mobility, invasion, and metastasis. It also stimulates natural killer and T cells, which can eliminate cancer cells.^[58]^ TGF-β1 has 2 roles in cancer: it suppresses tumors in the early stages of development and promotes the progression of many cancer types in the later stages.^[59]^ IFNG is considered a central player in antitumor activity; it exerts its anti-cancer effects by impacting both tumor and immune effector cells.^[60]^ TNF-α is a tumor-promoting factor associated with all tumorigenic stages in many cancers.^[61]^ All these cytokines are expressed in different cells in TME and can modulate tumor growth. Downregulated hub genes are positively correlated with these cytokines, which means these hub genes may be linked with cytokine production pathways.^[62,63]^ IL-6 and IL-33 negatively correlate with *COL11A1*, that is, IL-6 and IL-33 play a protective role in cancer development.

Furthermore, the positive correlations seen between downregulated hub genes and immune cells may suggest that these genes control the invasion of immune cells.^[64]^ A positive relationship between upregulated genes and immune cell infiltration implies that the higher expression of these genes contributes to the increased presence of immune cells in TME. These upregulated genes may encode or activate proteins that act as chemoattractants, cytokines, or other factors that recruit and activate immune cells.

Higher expression levels of *BGN*, *FN1*, *COL11A1*, and *SPTBN1* are linked to poorer overall survival in breast cancer patients is noticeable. These genes are linked with cytoskeletal proteins and extracellular matrix components, suggesting potential roles in TME remodeling and metastasis. Their overexpression might contribute to increased tumor aggressiveness, invasion, and resistance to treatment, all of which can negatively impact patient survival. The reduced expression of *CAV1, CXCL12,* and *INHBA* genes correlated with lower overall survival is also intriguing. *CAV1* is known for its tumor-suppressive properties and roles in regulating signaling pathways. The downregulation of *CAV1* may result in increased tumor growth and progression. *CXCL12* plays a crucial role in immune cell trafficking and tumor suppression. Reduced *CXCL12* levels may lead to compromised immune responses within the TME. INHBA encodes TGF-β superfamily members, and its decreased expression could affect TGF-β signaling, which is known to be involved in tumor progression, angiogenesis, and immune evasion.

While our study identified key DEGs, hub genes, cytokines, and regulatory networks involved in breast cancer, due to the nature of bioinformatics work, external experimental validation using RT-PCR is necessary for confirming the expression of DEGs. Integration of multi-omics data such as proteomics and metabolomics, would potentially provide a more comprehensive view of the TME.

There are several limitations in this study. First, all datasets used in this study were microarray-based, which is less powerful than RNA-Seq. Although the datasets were combined to identify shared DEGs, differences between studies may still arise due to variations in sample collection from different geographic regions, sample handling, microarray platforms, study designs, technical replicates, analytical techniques, and biological variability. We did not use any artificial intelligence-based approach such as machine learning or deep learning, which are now promising fields for the early detection of breast cancer.^[65]^

## 5. Conclusion

This comprehensive study on breast cancer highlights the identification of 36 DEGs across 10 datasets, suggesting common gene expression patterns pivotal in the disease. The analysis reveals that these DEGs, including tumor suppressors and potential biomarkers, play significant roles in cancer initiation, progression, and metastasis. The study’s extensive bioinformatics approach, incorporating RNA sequencing, pan-cancer analysis, and functional enrichment, unveils the complex interplay of these genes in the TME, particularly emphasizing their involvement in extracellular matrix remodeling and cancer cell proliferation. The PPI and epigenetic regulation analyses further underscore the importance of these genes as potential therapeutic targets. The study also elucidates the roles of transcription factors and miRNAs in gene regulation, adding depth to our understanding of breast cancer’s molecular landscape. The unique expression patterns of cytokines in the TME, their correlation with hub genes, and their impact on patient survival provide valuable insights for developing targeted therapies. In summary, this research offers a profound understanding of the molecular landscape of the breast cancer microenvironment, which can help improve diagnostic, prognostic, and therapeutic strategies. However, experimental validation is required to confirm the functional roles and biological relevance of the identified DEGs.

## Author contributions

**Conceptualization:** A.H.M. Nurun Nabi.

**Data curation:** Abdullah Al Noman.

**Formal analysis:** Abdullah Al Noman, Abdullah Al Saba.

**Investigation:** Abdullah Al Noman, Abdullah Al Saba, Mohammad Sayem.

**Methodology:** Abdullah Al Noman.

**Resources:** A.H.M. Nurun Nabi

**Software:** Abdullah Al Noman, Abdullah Al Saba.

**Supervision:** Tahirah Yasmin, A.H.M. Nurun Nabi.

**Visualization:** Abdullah Al Noman.

**Writing – original draft:** Abdullah Al Noman, Abdullah Al Saba, Mohammad Sayem.

**Writing – review & editing:** Tahirah Yasmin, A.H.M. Nurun Nabi.
